# Biocompatible Nanocomposite Coatings Deposited via Layer-by-Layer Assembly for the Mechanical Reinforcement of Highly Porous Interconnected Tissue-Engineered Scaffolds

**DOI:** 10.3390/bioengineering9100585

**Published:** 2022-10-20

**Authors:** Aoife McFerran, Mary Josephine McIvor, Patrick Lemoine, Brian J. Meenan, Jonathan G. Acheson

**Affiliations:** Nanotechnology and Integrated Bioengineering Centre (NIBEC), School of Engineering, Ulster University, Belfast BT15 1ED, UK

**Keywords:** layer-by-layer assembly, nanocomposite coating, bone tissue scaffold, biocompatible, compressive elastic modulus

## Abstract

Tissue-engineered (TE) scaffolds provide an ‘off-the-shelf’ alternative to autograft procedures and can potentially address their associated complications and limitations. The properties of TE scaffolds do not always match the surrounding bone, often sacrificing porosity for improved compressive strength. Previously, the layer-by-layer (LbL) assembly technique was used to deposit nanoclay containing multilayers capable of improving the mechanical properties of open-cell structures without greatly affecting the porosity. However, the previous coatings studied contained poly(ethylenimine) (PEI), which is known to be cytotoxic due to the presence of amine groups, rendering it unsuitable for use in biomedical applications. In this work, poly(diallydimethylammonium chloride) (PDDA)- and chitosan (CHI)-based polyelectrolyte systems were investigated for the purpose of nanoclay addition as an alternative to PEI-based polyelectrolyte systems. Nanocomposite coatings comprising of PEI, poly(acrylic acid) (PAA), Na+ montmorillonite (NC), PDDA, CHI and sodium alginate (ALG) were fabricated. The coatings were deposited in the following manner: (PEI/PAA/PEI/NC), PEI-(PDDA/PAA/PDDA/NC) and (CHI/ALG/CHI/ALG). Results from scanning electron microscopy (SEM) and energy-dispersive X-ray (EDX) analyses demonstrated that the nanoclay was successfully incorporated into each polymer bilayer system, creating a nanocomposite coating. Each coating was successful at tailoring the elastic modulus of the open-cell structures, with polyurethane foams exhibiting an increase from 0.15 ± 0.10 MPa when uncoated to 5.51 ± 0.40 MPa, 6.01 ± 0.36 MPa and 2.61 ± 0.41 MPa when coated with (PEI/PAA/PEI/NC), PEI-(PDDA/PAA/PDDA/NC) and (CHI/ALG/CHI/ALG), respectively. Several biological studies were conducted to determine the cytotoxicity of the coatings, including a resazurin reduction assay, scanning electron microscopy and fluorescent staining of the cell-seeded substrates. In this work, the PDDA-based system exhibited equivalent physical and mechanical properties to the PEI-based system and was significantly more biocompatible, making it a much more suitable alternative for biomaterial applications.

## 1. Introduction

With over 2 million bone grafts performed annually worldwide, bone defect reparation is rapidly becoming one of the most common procedures in bone regenerative medicine [1]. Bone defects can occur due to a wide range of conditions, such as various diseases [2], trauma, infection [1,3] or non-unions (failure of bone to heal) [4,5]. Non-unions, also known as critical-sized defects, do not heal naturally on their own throughout the course of a patient’s lifetime and thus require additional procedures to prevent more extensive damage [6]. As autograft procedures maintain the natural osteogenic qualities and exhibit sufficient structural stability [7,8], they are currently the gold standard for non-union repair. However, this grafting method has several drawbacks, including the introduction of a second surgery site, elevated rates of post-procedure infection [9,10], donor site complications [11] and limited grafting material [12]. An engineered ’off-the-shelf’ solution would address many of these complications. Tissue-engineered (TE) scaffolds have been shown to facilitate the regeneration of damaged tissue [7] and provide an effectively limitless material that can potentially reduce the healing time and limits the risk of infection [13]. Such TE scaffolds must match the physio-mechanical requirements that are exhibited by bone [14,15], including an elastic modulus ranging from 0.05–0.5 GPa [16] and a porosity range of 75–85% [17]. However, the porosity of the scaffold is often sacrificed in order to obtain sufficient mechanical properties. Layer-by-layer (LbL) assembly is a well-established technique for the application of thin film coatings, which achieves a coating by depositing alternating layers of oppositely charged materials onto a substrate. The versatility of this method [18] allows for the use of many different materials and the incorporation of various functionalities. Poly(ethylenimine) (PEI), poly(acrylic acid) (PAA) and cloisite Na+ montmorillonite (NC) coatings have been utilised previously in LbL assembly by Podsiadlo et al. and Ziminska et al. for the mechanical reinforcement of open-cell structures. Through the deposition of this mechanically robust thin film, it is possible to tailor the mechanical properties of a porous structure without excessively sacrificing its porosity [19]. Although these coatings can be used to tailor the mechanical properties of structures, the cytotoxic nature of PEI [20] means that their use as a biomaterial is significantly limited. In this work, the incorporation of nanoclay into other polymer layers, including PDDA/PAA and CHI/ALG, to create nanocomposite thin films was investigated. These polyelectrolytes exhibit an exponential growth regime under specific conditions [21,22], similar to PEI/PAA, and thus offer appropriate alternatives for researchers to explore. Coatings deposited on open-cell foams and glass slides were characterised to assess their unique properties under ambient conditions, and the cytotoxicity of the coatings was investigated. The coating topography/morphology, biocompatibility and mechanical integrity were examined to understand the resulting differences between the PEI substitutes.

## 2. Materials and Methods

### 2.1. Substrate and Polyelectrolyte Preparation

Glass slides (Brand, Wertheim am Main, Germany) and polyurethane (PU) foams (The Foam Shop, Taunton, UK) were prepared for pre-coating by washing in 1 M of NaOH (Sigma Aldrich, St. Louis, MO, USA) for 10 min and rinsing with 18.2 MΩ deionised water from a Milli-Q Purification System (Sigma Aldrich, St. Louis, MO, USA). Solutions of 1 wt.% of PEI, PAA and PDDA and 0.5 wt.% NC and sodium alginate (ALG) were prepared in 18.2 MΩ deionised water. Then, 0.5 wt.% chitosan (CHI) was prepared in lactic acid solution (1% *v*/*v*). The PEI, PDDA, ALG, CHI and lactic acid solution were all purchased from Sigma Aldrich (St. Louis, MO, USA), while NC was obtained from BYK Additives (Wesel, Germany) and PAA was purchased from Alfa Aesar (Lancashire, UK). The pH values of the PAA, PDDA and CHI were adjusted from ~2 to 8, ~6 to 4 and ~4 to 7, respectively, via the addition of 1 M of NaOH. The pH of ALG was adjusted from ~6 to 3 with 88% conc. lactic acid, and the pH levels of PEI and NC were left unadjusted. The pH levels were adjusted to achieve a specific growth regime of the polyelectrolytes. In this work, an exponential growth was desired. Samples assembled with PDDA were subjected to PEI solution for 30 s before coating to maximise the adhesion of the first layer.

### 2.2. Coating Method

In this work, the dipping method was utilised to coat the glass slides, as it offers a good reproducibility of the film formation and the precise control of the coating thickness [23]. The PU foams were coated using a unique solution replacement chamber. For the experimentation, both the microscopic glass slides and polyurethane (PU) foam were coated to represent 2D and 3D substrates, and the glass slides were coated using a customised dipping robot setup, Table 1, with the following coating sequences:

The PU foam substrates were coated using a solution replacement chamber setup, where the foam was placed into the chamber and subjected to each of the cationic, anionic and NC solutions for 30 s. In between the application of each polyelectrolyte solution, the solution in the chamber was replaced by 18.2 MΩ DI water for 6 intervals of 5 s. Polymer-clay nanocomposite coatings were then assembled in a sequence of (PEI/PAA/PEI/NC)_n_, PEI-(PDDA/PAA/PDDA/NC)_n_ and (CHI/ALG/CHI/NC)_n_, where n represents 15, 30, 45 and 60 quad layers (QLs). Moving forward, the (PEI/PAA/PEI/NC), PEI-(PDDA/PAA/PDDA/NC) and (CHI/ALG/CHI/NC) coatings will be referred to as PEI, PDDA and CHI, respectively. For the in vitro studies, square glass cover slips (15 × 15 mm, double thickness (0.19–0.23 mm), AGL 46S15-2, Agar Scientific, Essex, UK) were prepared and coated utilising the same method as described above.

### 2.3. Zeta Potential

The charge of all the polyelectrolyte solutions was determined by a Zetasizer NanoZS laser diffractometer through phase analysis light scattering (Malvern instruments Ltd., Malvern, UK), Table 2. The resulting polyelectrolyte solutions were adjusted and measured to maintain the following charges:

### 2.4. Scanning Electron Microscopy with Energy-Dispersive X-ray (SEM-EDX)

SEM was utilised to examine the coating topography of the 2D glass substrates deposited with the PEI, PDDA and CHI coatings. Prior to examination, the samples were coated with a conductive layer of gold palladium using a sputtering system (Emitech K500X, Quorum Technologies, Lewes, UK) at 25 mA for 2 min. Micrographs were collected by a Hitachi SU5000 field emission scanning electron microscope (SEM) (Hitachi, Tokyo, Japan) under a high vacuum in the secondary electron (SE) mode, with an accelerating voltage of 4 keV, to maximise the surface sensitivity. An energy-dispersive X-ray (EDX) analysis was then conducted under the same conditions. A total of 3 areas of each surface were examined at a resolution of 1024 × 1024, and the analysis was performed using the Aztec software (Oxford Instruments, Abingdon, UK). This analysis was performed at 4 keV, which corresponds to an electron range of approximately 600 nm in a polymeric material of a similar density. Therefore, only the coating, specifically the NC, was detected, without obtaining a contribution from the underlying glass substrate.

### 2.5. Stylus Profilometry

To assess the coating thickness, half of the glass slide was masked with Scotch tape (3 M, Berkshire, UK) so that the step height between the coated and masked, uncoated section could be measured. Stylus profilometry scans were performed, Table 3, using a Bruker Dektak XT (Bruker, Billerica, MA, USA) and a diamond conical tip with the following parameters:

Three samples from each coating type were assessed, and five parallel scans of the surface were taken for each sample. Samples were placed in a desiccator in a humidity of ~10% for 24 h to ensure that the coatings were tested in the same conditions.

### 2.6. Coating Density

The coated and uncoated glass slides were weighed using a microbalance (Radwag, Radom, Poland) with an accuracy of 0.001 mg. Volumetric measurements of the coating were determined by calculating the coating area and thickness. The coating area was defined by optical microscopy and the thickness measurements were retrieved by line profilometry. The density was then calculated by:

(1) $$Density\;=\;\frac{mass\;\left(g\right)}{volume\;\left({\mathrm{cm}}^{3}\right)}$$

### 2.7. Micro-Computed Tomography (CT)

A SkyScan 1275 (Bruker, Billerica, MA, USA) system was utilised for the micro-CT analysis, conducted with an operating voltage and current of 25 kV and 125 µA, respectively, and a pixel size of 8.5 µm. The samples were scanned and, using the NRecon software (Bruker, Billerica, MA, USA), we reconstructed the rotational slices before thresholding so as to determine the presence of the material. All the scans were analysed with the same reconstruction and thresholding settings to obtain comparable results. A volumetric bitwise analysis was performed for each sample to determine the porosity and interconnectivity using the CTAn software (Bruker, Billerica, MA, USA).

### 2.8. Instron Mechanical Testing

Compressive mechanical testing was conducted using an Instron machine (Instron 5660 series, Norword, MA, USA) and a 50 N load cell. The coated foams were placed in a desiccator for up to 48 h prior to mechanical testing to ensure that the coatings were completely dry. The samples were compressed at a rate of 2 mm/min to obtain a total displacement of 0.6 mm. Stress–strain curves were generated, and the elastic modulus was calculated using the linear (elastic) region of the stress–strain curve, as per ISO844.

### 2.9. Cell Culture

Cell studies were completed using a ‘medium’ comprised of McCoy’s 5A medium with L-glutamine supplemented with 10% foetal bovine serum and 1% penicillin/streptomycin mix (5000 units:5 mg/mL, respectively) under standard incubation conditions (a humidified incubator at 37 °C with 5% CO_2_). An immortalised human osteosarcoma cell line, U-2 OS (HTB-96, ATCC, Manassas, VA, USA), was chosen as a ‘model’ for human bone cells over the other commonly used osteosarcoma cells, such as MG-63s and Saos-2s, because the follow-on biological studies aimed to investigate the cell maturation, and the authors wanted to ensure that the cell line used for the preliminary biological studies demonstrated maturation characteristics that can mineralise to form bone-like nodules in vitro. The authors previously reported such cell maturation results with this cell line using microscopy techniques and Raman spectroscopy [24]. The cells were monitored daily using an inverted microscope (Nikon Eclipse TS100, Nikon, Amsterdam, The Netherlands) and passaged every 3–4 days to ensure that confluency was maintained at ~70%. Unless otherwise stated, all reagents were supplied from Sigma Aldrich, Merck, UK. All cell culture and associate biological assays were performed aseptically in a class 2 biosafety cabinet (Bioquell UK Ltd., Andover, UK).

### 2.10. Cell Count and Seeding

Prior to conducting the in vitro cell assays, each substrate type was individually wrapped in triple-layered aluminium foil and tightly sealed with autoclave tape. To remove any microbial load, the substrates were then placed in a dry oven (OV-12, Thermo Fisher Scientific, Waltham, MA, USA) for 4 h at 160 °C. The sterile substrates were placed in a class 2 biosafety cabinet (Bioquell UK Ltd., Andover, UK) until they were transferred to sterile 12-well tissue culture plastic plates. The cell suspension concentration, at day 0 and passage number 11, was determined as per the manufacturer’s protocol using an automated cell counter (TC20, Bio-Rad Laboratories Ltd., Watford, UK). The suspension was standardised as 4 × 10^5^ cells per ml, with 50 µL (20,000 cells per substrate) centrally pipetted onto each substrate and incubated for 2 h under standard conditions. An additional 1950 µL of medium was then pipetted into each well and the plates incubated until each time point (days 2, 3 and 7). The tissue culture plastic (TCP) controls were represented by the as-received square glass cover slips, where negative controls contained medium only and positive controls contained cell suspension and medium. These were treated under the same conditions as the test substrates, as previously stated.

### 2.11. In Vitro Cell Assay to Measure Cell Viability

The cell viability at days 2, 3 and 7 was evaluated using the resazurin reduction (RR) assay, an assay commonly used to monitor and quantify the number of viable cells in a sample/substrate at a particular timepoint based on the principle that the mitochondrial respiratory chain of any viable cells will reduce the oxidised non-fluorescent blue resazurin to fluorescent red resorufin. Resazurin salt (ab145513-5g, purity > 80%, abcam, Cambridge, UK) was dissolved in 0.01 M of phosphate-buffered solution (PBS) to 0.30 mg/mL and sterile-filtered using a 0.2 µm filter into a light-protected container. Aliquots were then prepared and stored, ensuring light protection. The substrates and their medium were placed in 12-well plates and 100 µL of RR solution was added to each well. The plates were wrapped in aluminium foil, rotated at 20 RPM for 5 min at room temperature and then incubated for 4 h. Once incubated, each well was thoroughly mixed, and 3 × 100 µL of the contents per well was transferred to a black 96-well plate, again ensuring light protection until the plate was read. The fluorescence was recorded using 560 nm excitation/590 nm emission filter set with a Tecan Spark multi-mode plate reader (Tecan Group Ltd., Seestrasse, Switzerland).

### 2.12. Scanning Electron Microscopy of U-2 OS Cells

The medium was aspirated from the wells of each tissue culture plastic (TCP) control and test substrate, and the wells were washed twice with 0.01 M PBS followed by 3 washes of deionised water (to minimise any PBS crystallisation within the imaged cells), with each well aspirated between each wash. Following this, 2.5% of glutaraldehyde (in deionised water) was added for 45 min at room temperature and then aspirated. An alcohol series of increasing ethanol concentrations were used to gradually dehydrate the samples. Concentrations of 25, 50, 75, 90 and 100% were added consecutively for 8 min at room temperature, with the wells aspirated between each concentration. Once aspirated, a 1:1 ratio of ethanol and hexamethyldisilane (HDMS) was added and incubated at room temperature for 8 min. The wells were aspirated, and 2–3 drops of HMDS were added in order to thoroughly dry the samples at room temperature overnight. The coated square glass cover slips were coated with a conductive layer (~18 nm) of gold palladium using a sputtering system (Emitech K500X, Quorum Technologies, Lewes, UK) at 25 mA for 2 min before examination by SEM (Hitachi SU5000 instrument, Hitachi, Buckinghamshire, UK).

### 2.13. 4′,6-Diamidino-2-Phenylindole (DAPI) Staining

Medium from each well was aspirated, and the substrates washed twice with 0.01 M PBS. The wells were aspirated, and 4% paraformaldehyde (PFA, in 0.01 M PBS) was added for 8 min at room temperature. The substrates were washed twice with 0.01 M PBS prior to staining the cells with 300 nM 4′,6-diamidino-2-phenylindole (DAPI) in 0.01 PBS for 10 min at room temperature. Prior to imaging, the substrates were washed 3 times with 0.01 M PBS. The substrates were viewed and imaged using a 10× objective, and any fluorescently stained ‘blue’ nuclei were automatically counted using a wide-field fluorescent microscope and its associated software (Zeiss Axio Imager and Zen software, Zeiss GmbH, Oberkochen, Germany). At least 5 random areas per DAPI-stained substrate were imaged using Colibri 7 LED illumination at an excitation wavelength of 405 nm for 820 milliseconds. Images were captured at a resolution of 1024 × 1024 pixels.

### 2.14. Statistical Analysis

The statistical analysis (*n* = 6) was performed using GraphPad Prism version 9.3.1 for Windows (GraphPad Software, San Diego, CA, USA). Ordinary one-way analysis of variance (ANOVA), including the Brown–Forsythe and Bartlett’s tests, were applied to test for statistically significant differences at each timepoint, with a value of *p* < 0.05 considered statistically significant. Any significant difference between the scaffold types was determined (and plotted) using Tukey’s multiple comparisons test, with a value of *p* < 0.05 taken as statistically significant.

## 3. Results

**Figure 1:**
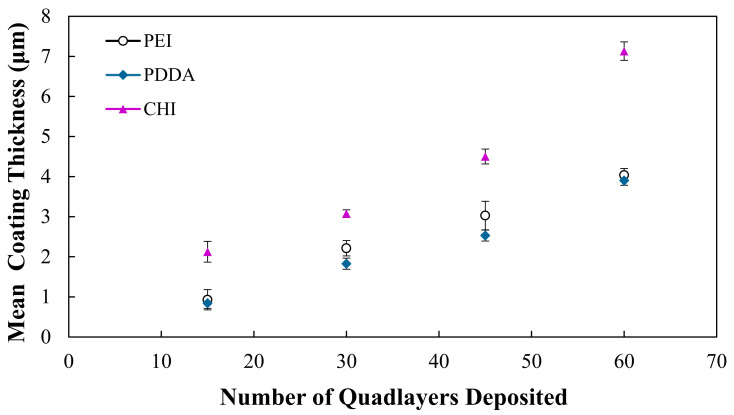
Mean coating thicknesses of PEI, PDDA and CHI coatings assembled with 15, 30, 45 and 60 QLs on glass microscopic slides.

As shown in Figure 1, the thickness of the coatings increases with the number of QLs deposited, and this is observed for all coating types on the glass microscopic slides. Throughout the deposition process, it can be observed that the growth of the PEI and PDDA coatings is quite similar, with both coating types reaching an approximate thickness of 4 μm at 60 QLs. The CHI coating maintains a thicker coating, and at 60 QLs it reaches a thickness of 7 μm.

The mean densities of the PEI, PDDA and CHI coatings deposited with 15 and 60 QLs are shown in Table 4. At 15 and 60 QLs, the PEI and PDDA coatings exhibit similar densities, whereas the CHI coating exhibits a slightly higher density.

**Figure 2:**
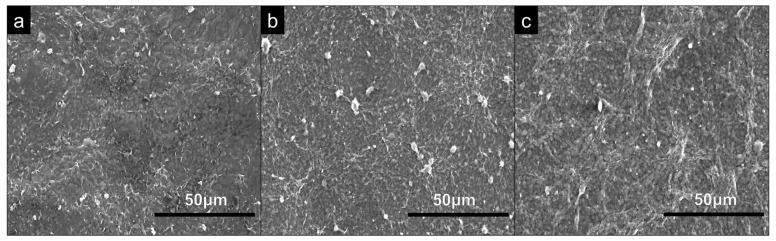
SEM analysis of 60 QL (**a**) PEI, (**b**) PDDA and (**c**) CHI coatings on glass microscopic slides examined at a magnification of ×1k.

SEM analysis was conducted on the substrates to assess any differences in topography across each of the coating types. From SEM micrographs, shown in Figure 2, all coating types show visible similarities in topography and surface features.

**Figure 3:**
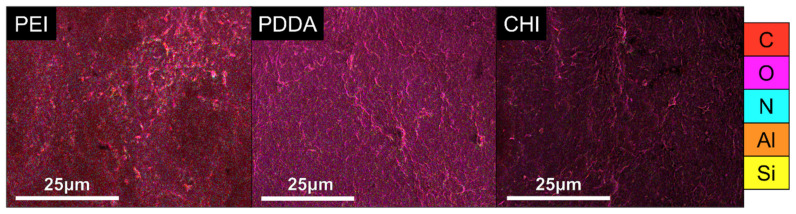
EDX maps of PEI, PDDA and CHI coatings where carbon, oxygen, nitrogen, aluminium and silicon are represented by C, O, N, Al and Si, respectively.

Results from the EDX analysis confirm that NC was successfully incorporated into each of the coatings. The major constituents of the NC include silicon and aluminium, and these elements are not observed in the polymers utilized in this work, thus providing an indication of the elemental composition of NC. It is evident from Table 5 that silicon and aluminium are present across all coating types. The EDX maps of the coating constituents are shown in Figure 3.

**Figure 4:**
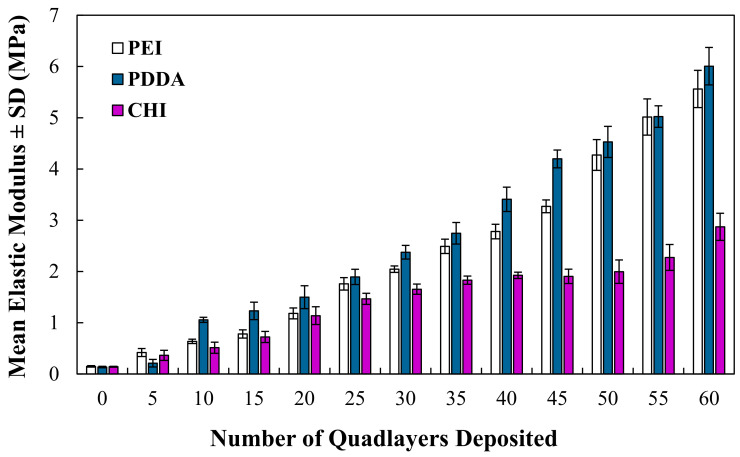
Mean elastic modulus ± SD of the coated foams as a function of the quad layers deposited.

The mechanical properties, derived from compressive testing, determine that the elastic modulus of the coated foams increases as a function of the number of QLs deposited, as shown in Figure 4. The modulus of the open-cell foams increased from 0.15 ± 0.10 MPa when uncoated to 5.51 ± 0.40 MPa, 6.01 ± 0.36 MPa and 2.61 ± 0.41 MPa for the PEI, PDDA and CHI coatings deposited with 60 QLs, respectively. The PEI and PDDA coatings exhibit comparable increases in the modulus throughout testing, although the CHI-coated foams exhibit a notably lower modulus, although, overall, this increases with the number of QLs deposited.

The porosity was assessed for both the uncoated and coated PU foams, and the porosity of an uncoated foam substrate was determined to be 98.67%. As the number of QLs deposited increased from 15 to 60, there was a slight decrease in the porosity exhibited by the coated foams (Table 6). The overall decrease in the porosity was 6.98%, 4.91% and 6.64% for the PEI, PDDA and CHI coatings, respectively. All three coating types demonstrated similar results, meaning that the PDDA and CHI coatings do not negatively impact the porosity any more than the PEI coating.

**Figure 5:**
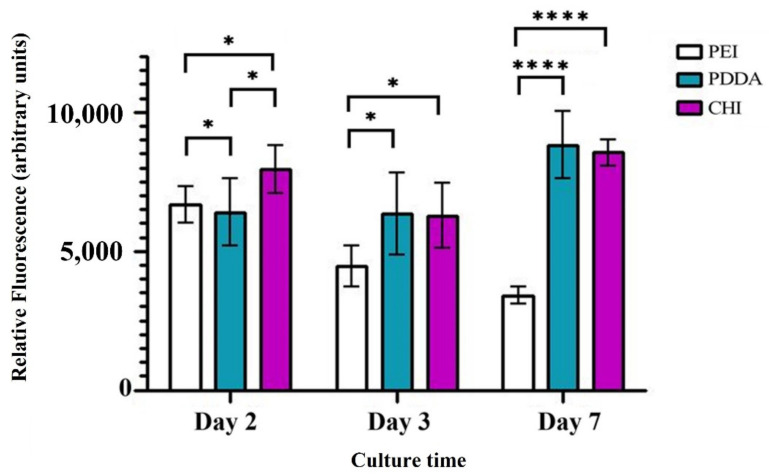
Resazurin reduction assay results as an indication of the U-2 OS cell viability upon exposure to the PEI, PDDA and CHI coatings on square glass cover slips, cultured under standard conditions for 2, 3 and 7 days. Each scaffold type was tested in triplicate. Any statistical significance is shown where *p* < 0.05. Increasing significance is represented by an increasing number of asterisks (* *p* < 0.05 and **** *p* < 0.0001.

There is a decreasing trend in the cell viability of the PEI coating as the culture time increases; however, this was not shown for the PDDA and CHI coatings, as seen in Figure 5. Reviewing the results of day 7, as the longest day of culture, it is apparent that the PEI coating has a considerably lower cell viability, with statistically significant differences (*p* < 0.0001) observed between PEI and PDDA, and the PEI and CHI coatings. There was no statistically significant difference between the PDDA and CHI coatings at day 7.

**Figure 6:**
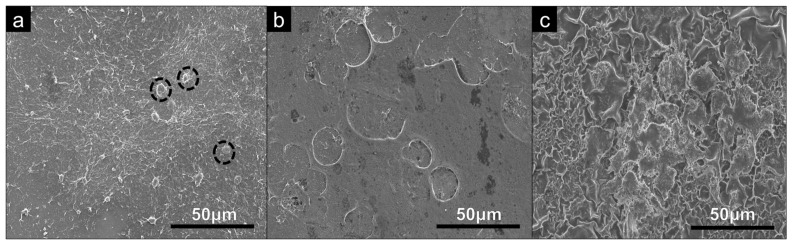
SEM analysis of U-2 OS cells seeded on square glass cover slips coated with (**a**) PEI coating, (**b**) PDDA coating and (**c**) CHI coating, examined at a magnification of ×1k and cultured under standard conditions for 7 days.

The adhered U-2 OS cells on the PDDA and CHI coating surfaces were examined by SEM at day 7 to provide qualitative information on their morphologies and osteoblast-like properties, as shown in Figure 6. These features are most evident in the PDDA and CHI coatings, shown in Figure 6b,c. The cells on these substrates exhibit a flattening morphology and, on further examination of the PDDA coating, we can see that the cells have potentially formed a film on this surface. The highlighted U-2 OS cells seeded onto the PEI coating maintain a rounded, almost spherical morphology and are also notably smaller at day 7 of the culture period. Spherical cells suggest that the filopodia do not successfully probe and are not attached to this surface, displaying an effective decrease in the cell viability.

**Figure 7:**
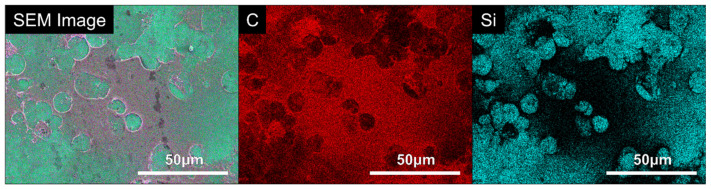
EDX analysis of the U-2 OS film formed on the square glass cover slips coated with the PDDA coating.

EDX was utilised for further analysis because the film formed on the surface of the PDDA coating was cellular. The elemental composition of the cellular film was examined, as shown in Figure 7, with the presence of silicon linked to the coating, due to its presence in the nanoclay layers, and carbon indicating a cellular material. EDX analysis confirmed that silicon was much less abundant in the areas of the cellular film on the surface of the substrate. Moreover, the high intensity of the carbon present on the film further corroborates that it is a cellular material.

**Figure 8:**
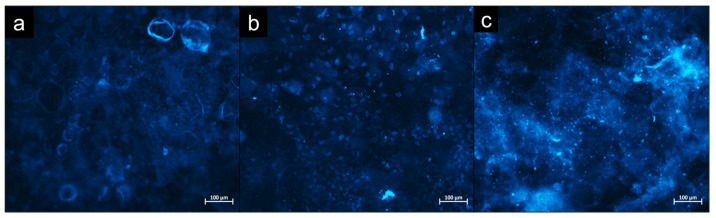
DAPI staining analysis of U-2 OS cells seeded on (**a**) PEI, (**b**) PDDA and (**c**) CHI coatings cultured under standard conditions for 7 days.

Once the DAPI staining was completed, the samples were analysed at days 2, 3 and 7 utilising fluorescence microscopy. Figure 8 displays the results from day 7. It is evident that there are no stained nuclei present on the PEI coating; however, there are numerous stained nuclei present on the PDDA and CHI coatings. The polyelectrolytes used to manufacture each of the coatings can act as an effective reservoir for reagents [25]. Therefore, conducting a quantitative analysis of the number of nuclei was not viable, as it was possible that some of the DAPI stain was contained within the coating, causing background fluorescence. However, the result of the qualitative analysis retrieved in this study was still representative of cytotoxicity.

## 4. Discussion

Nanocomposite coatings comprising of (PEI/PAA/PEI/NC) have been widely investigated in the literature for the purpose of mechanical reinforcement [19,26]. The physico-mechanical properties of this PEI-containing coating provide a useful baseline for the novel PDDA and CHI coatings that are investigated in this study. Previously, the PEI coatings were found to be fabricated as a function of the number of QLs deposited, and this work demonstrated that the PDDA and CHI coatings behave in the same manner, as shown in Figure 1, with gradual increases in the thickness throughout the deposition process. The PEI and PDDA coatings exhibit a similar coating thickness and overall growth increase from 15 to 60 QLs, whereas the CHI coating is significantly (*p* < 0.0001) thicker. As shown in Figure 4, through the application of each of the coating types, it is possible to significantly improve the elastic modulus of the highly porous open-cell structures. Our results indicate that the modulus of the foams can be controlled by varying the number of QLs deposited, allowing for a desired modulus to be achieved by altering the number of deposited QLs. As the design and properties of TE scaffolds are paramount to their application and achievement of a biomimetic structure suitable to cancellous bone [27], it is crucial that any coating deposited in order to add functionality does not drastically reduce the porosity of a potential scaffold. In this work, foams were coated with up to 60 QLs, and the porosity was assessed after every 15 QLs, as the predicted the foams exhibited a slight decrease in porosity with the increasing coating thickness. For the PEI and PDDA coatings, it was possible to significantly improve the mechanical properties of the foam, reaching a modulus of up to 6 MPa with just 60 QLs, whilst not excessively sacrificing the porosity of the structure. It has been demonstrated previously in the literature that the PEI coating can illustrate the desired mechanical properties (0.05–0.5 GPa [16]) required to tailor a potential bone tissue scaffold [19]. Whilst exhibiting a similar coating thickness, mechanical properties and porosity to the PEI coating, the PDDA coating could be just as useful for tailoring the mechanical properties. PEI is a polycation that consists of numerous amine groups (NH_2_) and two carbon aliphatic spacers (CH_2_CH_2_). Several studies have indicated that PEI is cytotoxic, and this could be due to the fact that PEI induces mitochondrial-mediated apoptosis. Moreover, PEI has also been shown to facilitate the reduction in the mitochondrial membrane potential and impairment of mitochondrial respiration, and it also induces its swelling [20,28,29]. The decreasing cell viability of the PEI coatings shown in Figure 5 suggests that the PEI, as a main constituent of the coating, causes a cytotoxic response to the U-2 OS cells seeded on the surface of this coating. Neither the PDDA nor CHI coating induced this response, and it has been suggested that the quaternary ammonium groups in PDDA exhibit a lower cytotoxicity compared to the primary amine groups in PEI [30]. As detailed earlier, chitosan was prepared in an acidic aqueous solution, which, in turn, promotes the protonation of the NH_2_ groups. The subsequent changes in the chitosan structure [31,32] may have a favourable influence on cytotoxicity and, therefore, may result in a higher cell viability than that of the PEI coatings. It is evident in Figure 6b,c and further indicated by Figure 7 that, at day 7, the PDDA and CHI coatings support the cell attachment and growth of the U-2 OS cells. The filopodia of the cells extending to the coatings indicate that the cells favour the surfaces exhibited by PDDA and CHI, facilitating their attachment. The cell flattening morphology was also demonstrated. This has been noted previously in the literature, and it has been suggested that it is due to the fact that the cells adhere more securely to the underlying substrate [33,34]. Cell attachment and flattening were not observed on the PEI coatings. In Figure 6a, the round spherical shape of the cells on this coating indicate the potential cytotoxicity of the underlying substrate. Fluorescent microscopy images, shown in Figure 8, detailing the stained nuclei of the U-2 OS cells, provide additional information on the cell viability and behaviour at day 7 of culture for each coating type. Stained nuclei were visible on both the PDDA and CHI coatings but not the PEI coating. Although cell counting was not viable, the qualitative analysis attained findings that corroborate the accompanying in vitro work.

## 5. Conclusions

In this work, nanoclay was successfully incorporated into PEI/PAA, PDDA/PAA and CHI/ALG polymer layers to create nanocomposite coatings of PEI/PAA/PEI/NC, PEI-(PDDA/PAA/PDDA/NC) and CHI/ALG/CHI/NC. Through the deposition of each of these coatings onto microscopic glass slides and open-cell structures, it was possible to tailor various properties as a function of the number of deposited QLs, including the coating thickness, porosity and elastic modulus. At 60 QLs, the PEI and PDDA coatings can be tailored to exhibit a modulus reaching over 5 MPa, whilst maintaining a similar porosity of approx. 88% and thickness of approx. 4 μm. The elastic modulus of the CHI coating was tailored to approx. 3 MPa at 60 QLs and demonstrated a porosity and coating thickness of approx. 89% and 7 μm.

This work has shown that the incorporation of nanoclay into polymer multilayers is not restricted to the use of PEI/PAA/PEI/NC for the purpose of mechanical reinforcement. This is because PEI-(PDDA/PAA/PDDA/NC) and CHI/ALG/CHI/NC coatings not only enhance the mechanical properties but also provide a system with significantly lower cytotoxicity. The lower cytotoxicity illustrated by the PDDA and CHI coatings was confirmed through a series of cell viability studies.

The PDDA coating explicitly presents as the most comparable with the physical and mechanical properties of the PEI coating. However, crucially, it offers a significantly higher biocompatibility. The findings in this work widen the potential use of these polymers and, subsequently, the nanocomposite coatings for the functionalisation of open-cell structures in biomedical applications, such as bone defect reparation.

## Figures and Tables

**Table 1:** Coating sequences for microscopic glass slides using the customised dipping robot.

Solution			Dip Time
PEI	PDDA	CHI	2 s
DI Rinse	DI Rinse	DI Rinse	3 s
PAA	PAA	ALG	2 s
DI Rinse	DI Rinse	DI Rinse	3 s
PEI	PDDA	CHI	2 s
DI Rinse	DI Rinse	DI Rinse	3 s
NC	NC	NC	2 s
DI Rinse	DI Rinse	DI Rinse	3 s

**Table 2:** Z-potential of the various polymer solutions under specific pH conditions.

Polyelectrolyte	Ζ-Potential (mV)	pH
PEI	7.63 ± 0.6	11
PAA	−25.87 ± 5.9	8
PDDA	21.5 ± 2.57	4.4
CHI	11.63 ± 0.76	7
ALG	−31.33 ± 2.20	3
NC	−28.61 ± 0.30	8

**Table 3:** Line profilometry parameters.

Stylus Type	Radius = 2 µm
Range	534 µm
Length	5000 µm
Duration	60 s
Stylus Force	3 mg
Profile	Hills and Valleys

**Table 4:** Mean coating densities of PEI, PDDA and CHI coatings on glass microscopic slides at 15 QL and 60 QL.

	15 QL		60 QL	
	Mean Density (g/cm^3^)	Std. Dev	Mean Density (g/cm^3^)	Std. Dev
PEI	1.12	0.26	1.29	0.29
PDDA	1.14	0.26	1.36	0.37
CHI	1.76	0.58	2.64	0.67

**Table 5:** EDX analysis conducted on glass slides deposited with 60 QL coatings.

	PEI Coating		PDDA Coating		CHI Coating	
Element	Atomic %	Std. Dev	Atomic %	Std. Dev	Atomic %	Std. Dev
C	23.33	0.38	49.63	0.57	38.23	0.85
O	36.97	0.68	31.03	4.88	39.53	0.86
N	25.67	1.01	5.93	3.17	5.07	0.25
Si	6.57	0.06	6.67	1.08	10.13	0.86
Al	2.97	0.50	3.05	0.64	4.03	0.15
Na	0.00	0.00	0.00	0.00	0.30	0.10

**Table 6:** Total calculated porosity of PEI, PDDA and CHI coated PU foams (*n* = 3) at 15 and 60 QLs.

	15 QL		60 QL	
	Total Porosity (%)	Std. Dev	Total Porosity (%)	Std. Dev
PEI	95.85	0.40	88.87	1.01
PDDA	93.55	1.35	88.64	0.60
CHI	95.92	0.68	89.28	0.51

## Data Availability

The data presented in this study is available on request from the corresponding author.
